# Advances in Micro- and Macrobiological Strategies for Pest Control in Berry Production Systems: A Critical Review

**DOI:** 10.3390/plants15010144

**Published:** 2026-01-04

**Authors:** Oscar Giovanni Gutiérrez-Cárdenas, Humberto Javier López-Macías, Kolima Peña-Calzada, Gerardo Arias-Robledo, Guadalupe Oyoque-Salcedo, Isaac Zepeda-Jazo, Pedro Damián Loeza-Lara, Martin Heil, Omar Fabián Hernández-Zepeda

**Affiliations:** 1 Genómica Alimentaria, Universidad de La Ciénega del Estado de Michoacán de Ocampo, Sahuayo 59103, Michoacán, Mexico; oggutierrez@ucemich.edu.mx (O.G.G.-C.); javierlopezmacias57@gmail.com (H.J.L.-M.); z_isaac@hotmail.com (I.Z.-J.); pdloeza@ucemich.edu.mx (P.D.L.-L.); 2 Faculty of Agricultural Sciences, José Martí Pérez University of Sancti Spíritus (UNISS), Sancti Spíritus 60100, Cuba; kolimapena@gmail.com; 3 Departamento de Ciencias de la Naturaleza, CUSUR, Universidad de Guadalajara, Enrique Arreola Silva 883, Ciudad Guzmán 49000, Jalisco, Mexico; gerardo.arias@academicos.udg.mx; 4 Investigación Aplicada-Fitosanidad-Driscoll’s, Avenida Miguel de la Madrid Hurtado #147 Centro, Ciudad Guzmán 49000, Jalisco, Mexico; 5 Investigación Aplicada-Fitosanidad-Driscoll’s, Ibramiento Sur #1620, Jacona 59833, Michoacán, Mexico; 6 Instituto Politécnico Nacional, Centro Interdisciplinario de Investigación para el Desarrollo Integral Regional (CIIDIR), Unidad Michoacán, Justo Sierra 28, Col. Centro, Jiquilpan 59510, Michoacán, Mexico; goyoque@ipn.mx; 7 Departamento de Ingeniería Genética, Centro de Investigación y de Estudios Avanzados del Instituto Politécnico Nacional-Irapuato, Irapuato 36500, Guanajuato, Mexico

**Keywords:** biological control agents, genetic enhancement, microbial–insect interactions, native isolates, sustainable agriculture

## Abstract

Berry crops such as strawberry *Fragaria* × *ananassa* (Weston), raspberry *Rubus idaeus* L., blackberry *Rubus ulmifolius* Schott, 1818, and blueberry *Vaccinium myrtillus* L. are economically and nutritionally valuable worldwide. However, the intensive use of synthetic pesticides for pest management in these crops has led to ecological imbalance, pest resistance, and negative effects on non-target organisms and human health. The integration of biological control agents into sustainable integrated pest management (IPM) systems represents an alternative. This review compiles and evaluates current advances in the application of baculoviruses (BVs), entomopathogenic fungi (EPFs), nematodes (EPNs), predatory mites (PMs), and parasitoid wasps (PWs) for pest suppression in berry crops. Emphasis was placed on their ecological interactions, host specificity, and compatibility within IPM frameworks. The combined use of micro- and macrobiological control agents effectively reduces key pest populations. However, field efficacy remains influenced by abiotic stressors such as UV radiation, temperature fluctuations, and chemical incompatibility. The integration of native micro- and macrobiological control agents of through conservation biological control (CBC) strategies can enhance sustainability in berry production systems. Future efforts should focus on formulation improvements, adaptive management under field conditions, and synergistic interactions among microbial and arthropod natural enemies.

## 1. Introduction

Berries, broadly defined as fruits derived from multiple achenes, encompass economically important crops such as strawberry *Fragaria* × *ananassa* (Weston) Duchesne ex Rozier (Rosaceae), blackberry *Rubus ulmifolius* Schott, 1818 (Rosaceae), raspberry *Rubus idaeus* L., 1753 (Rosaceae), and blueberry *Vaccinium myrtillus* L., 1753 (Ericaceae) [1,2]. Among these, strawberry has emerged as one of the most widely cultivated and consumed, representing a crop of high export value and a significant source of employment worldwide [3,4,5]. The global demand for berries continues to grow due to their nutritional and nutraceutical value; however, this expansion is constrained by increasing pest pressure and the strict regulatory frameworks imposed on export markets, particularly regarding pesticide residues [6].

Modern berry production systems are typically characterized by intensive monocultures, the use of plastic mulches, macrotunnels, and greenhouses, as well as irrigation systems designed to maximize yields. While these practices improve productivity, they simultaneously reduce genetic variability (given that most cultivars are propagated asexually) and favor the establishment of phytophagous pests [7,8]. These vulnerabilities, coupled with the widespread use of organosynthetic pesticides, have generated multiple challenges: the selection of resistant pest populations, detrimental effects on non-target organisms, contamination of ecosystems, and health risks for humans and animals [9,10].

Berries are attacked by a broad range of pests, including red spider mite *Tetranychus urticae* (Koch, 1836) (Trombidiformes: Tetranychidae), fall armyworm *Spodoptera frugiperda* Smith & Abbot, 1797 (Lepidoptera: Noctuidae), lygus bug *Lygus* spp. (Hemiptera: Miridae), thrips *Frankliniella occidentalis* (Pergande, 1895) (Thysanoptera: Thripidae), the Asiatic fruit fly *Drosophila suzukii* (Matsumura, 1931) (Diptera: Drosophilidae), black vine weevil *Otiorhynchus sulcatus* (Fabricius, 1775) (Coleoptera: Curculionidae), strawberry crown moth *Synanthedon bibionipennis* (Boisduval, 1869) (Lepidoptera: Sesiidae), and strawberry sap beetle *Lobiopa insularis* (Laporte de Castelnau, 1840) (Coleoptera: Nitidulidae) [11,12,13,14,15,16]. Conventional integrated pest management (IPM) programs, although widely promoted, are often criticized for acting as symptomatic “placebo-like” measures that mitigate visible damage without addressing the ecological drivers of pest outbreaks. Furthermore, organic berry production remains marginal in developing countries, and even in such systems, inputs of reduced-toxicity pesticides are still common [17]. Regardless of whether pesticides are of chemical or biological origin, differences in their efficacy depend on the target pest organism, the production system (open field or greenhouse), and the environmental context surrounding the crop [17]. However, the scientific literature indicates that synthetic chemical pesticides typically provide a rapid reduction of phytophagous pest populations, particularly under high infestation scenarios, due to their immediate mode of action and broad spectrum of activity. These characteristics have historically positioned them as the primary strategy for pest control in agricultural systems [18]. In contrast, biopesticides offer greater specificity and reduced environmental impact, making them well-suited for application within IPM programs in berry production that emphasize sustainability. However, their effects are generally slower than those of chemical controls [19]. Under this scenario, biotechnological interventions have emerged as complementary tools with high potential within IPM programs. Among these, the sterile insect technique (SIT) targeting *D. suzukii* has been identified as a highly specific alternative for the management of key pest species in berry cropping systems. In this regard, Hemer et al. [20] evaluated the effectiveness of SIT under commercial production conditions, assessing the biological quality of irradiated sterile males in terms of mating competitiveness, courtship behavior, flight performance, population suppression, and reduction of fruit damage in commercial raspberry fields. The results demonstrated that the SIT-based treatment performed comparably to conventional programs relying on season-long applications of synthetic chemical pesticides implemented by growers. Moreover, released sterile males exhibited levels of competitiveness similar to those of non-irradiated fertile males, resulting in reductions of up to 89% in wild female *D. suzukii* populations and approximately an 80% decrease in the number of larvae per harvested fruit, accompanied by a reduction in relative fruit losses of up to 58%. These limitations highlight the urgent need to expand the role of biological control (BC) within IPM frameworks, particularly through approaches that integrate both microbiological agents such as baculoviruses (BVs), entomopathogenic fungi (EPFs), entomopathogenic nematodes (EPNs), and macrobiological agents (predators and parasitoids). Furthermore, in the case of EPFs, certain strains have been genetically transformed to enhance their efficacy and dispersal capacity. For example, *Metarhizium anisopliae* (Metschn.) Sorokin, 1883 has been engineered to express scorpion toxin genes [21], thereby increasing its effectiveness against lepidopteran and hemipteran insects. Similarly, in *Beauveria bassiana* (Bals.-Criv.) Vuill., 1912, researchers have modified the expression of the non-ribosomal peptide synthetases (NRPSs), which are involved in the activation of genes that code for enzymes responsible for synthesizing secondary metabolites to maximize its insecticidal activity [22]. In the near future, genetically modified fungal strains are expected to be applied in agriculture under controlled conditions, potentially providing significant economic benefits [23,24,25,26]. In this context, Zhou et al. [2] conducted studies establishing highly efficient genome editing using CRISPR-Cas9 in *Fragaria vesca* L., 1753, demonstrating the stable transmission of targeted mutations through the germline, a critical prerequisite for the development of durable pest-resistance traits. Accordingly, the editing of key regulatory genes in the auxin signaling pathway (*TAA1* and *ARF8*) generated heritable mutations aimed at modifying physiological pathways involved in plant–insect interactions. Notably, plants carrying homozygous knockout mutations in ARF8 exhibited accelerated growth, a trait that could be indirectly exploited to reduce pest susceptibility through alterations in plant architecture and phenology. Collectively, these findings position CRISPR-Cas9 as a strategic tool for the development of strawberry crops with genetically based resistance to insect pests, with strong potential for integration into IPM programs.

In macrobiological control, the main factors affecting its efficacy and efficiency include the irrational use of organosynthetic pesticides [27], availability and stability of prey/hosts [28], intensive agricultural practices [29], intraguild competition and predation [30], low diversity of refuges and alternative resources [31], poorly planned mass releases [32], limited mobility and dispersal capacity [33], and socioeconomic factors [34]. In contrast to entomopathogens, a high level of efficacy has been demonstrated under controlled conditions, although their field performance is often constrained by adverse environmental factors such as temperature fluctuations, humidity, and UV radiation, which limit their persistence and activity [35,36]. Consequently, successful implementation requires strategies beyond inundative releases, including the conservation of NEs or conservation biological control (CBC), which promotes the maintenance and stability of beneficial organisms within agroecosystems [37,38]. In this context, the integration of micro- and macrobiological control agents offers a promising pathway to reduce reliance on organosynthetic pesticides, enhance the sustainability of berry production, and align with the demands of international markets that increasingly prioritize residue-free fruit [18,39].

Based on these considerations, this review critically evaluates recent advances in microbiological and macrobiological pest control strategies in berry cropping systems. By systematically integrating ecological, physiological, and technological perspectives, this work offers a novel, cross-disciplinary synthesis of the factors governing the effectiveness of parasitoids, predators, and entomopathogens. Importantly, the review goes beyond descriptive analysis by identifying underexplored mechanisms, methodological limitations, and priority research areas that are critical for optimizing the deployment of biological control agents. These insights provide a strategic framework to support their effective and sustainable integration into contemporary IPM programs for berry crops at a global scale.

## 2. Arthropod Pest Management in Berries: Conventional and New Options

Berries are affected by various phytophagous pests that considerably affect their production [16,40]. Predominantly, in global agroecosystems, the control of these arthropods relies exclusively on the use of organosynthetic pesticides because they are cheap, easy to apply, and generally more effective compared to biological pesticides [41,42]. Nevertheless, the increasing difficulty in controlling certain pest species as a result of evolved resistance, together with growing consumer demand for agroecologically produced crops, has driven the adoption and further development of microbial pesticides as alternative pest management tools [43,44]. This is reinforced by the fact that various mites, Diptera, Hemiptera, Lepidoptera and Thysanoptera pests extensively affect berry production, causing significant yield losses. In this context, entomopathogens have been described as microorganisms capable of invading and replicating within an insect host, subsequently disseminating to infect additional individuals [45], the most commonly used being BVs, EPFs, and EPNs [46,47,48]. Many of the most widely used biopesticides are already commercially available in some countries. Baculoviruses are biocontrol agents of insect pests, the most commonly used of which are found in the Baculoviridae family which comprises two genera: Nucleopolyhedroviruses (NPVs) and Granuloviruses (GVs) [49,50,51] with AcMNPV standing out for its ability to infect a wide range of lepidopteran pests [52]. For the baculoviruses, more than 600 hosts have been recorded due to their high host specificity [48,53].

**Figure 1:**
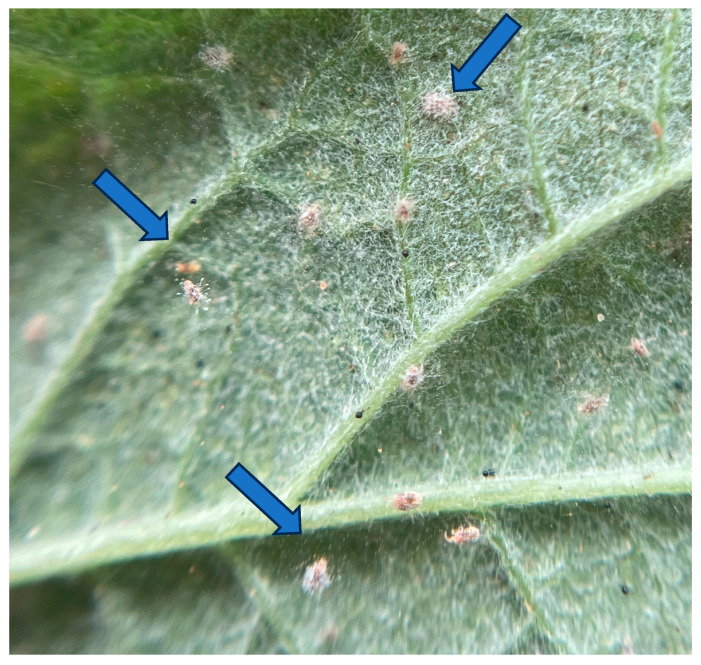
*Tetranychus urticae* infection caused by the EPF *C. fumosorosea* on a strawberry leaf. Image courtesy of author G.A.-R.

In the case of EPFs, the most widely used for their biocontrol capacity are *B. bassiana*, *Hirsutella thompsonii* F.E. Fisher, 1950, *M. anisopliae*, *M. rileyi* (Farl.) Kepler, S.A. Rehner & Humber, 2014, and *Cordyceps fumosorosea* (Wize) Kepler, B. Shrestha & Spatafora, 2017, being the first biological control agents registered for their ability to cause epizootics in phytophagous pests [17,54]. The scientific literature reports that more than 700 host species are included in various orders [55,56]. A study evaluated two native fungal isolates, *M. robertsii* and *B. bassiana*, originally isolated from maize (*Zea mays* L., 1753) and strawberry crops, respectively, by inoculating strawberry roots under field conditions to assess their efficacy in the management of *T. urticae*. The results obtained revealed a significant decrease in spider mite adults per strawberry leaflet (225.6 ± 59.3 and 206.5 ± 51.4) for the plants treated with *M. robertsii* and *B. bassiana*, whereas, in the control, there were 534 ± 115 spider mite adults present [57]. Based on the foregoing, Figure 1 illustrates a case of natural regulation of this pest population by the EPF *C. fumosorosea* under field conditions within a strawberry production system. This observation supports the hypothesis that natural enemies can play a significant role in the biological control of pests in agroecosystems, even in the absence of anthropogenic intervention.

## 3. Role of Entomopathogenic Nematodes in IPM Programs for Berry Production

In berry crops, EPNs represent a sustainable IPM strategy, as they encompass a wide range of species with high potential in biological control. As components of ecosystems, EPNs are important agents for regulating pest insect populations [58]. Furthermore, EPN-based bioinsecticides are highly effective, given that their commercial development has progressively increased over the past decades [59,60]. These agents are characterized as non-segmented, soft-bodied roundworms that act as obligate parasites of insects and measure approximately 0.5 mm in length [45]. They have been extensively employed in IPM programs worldwide.

In particular, the current scientific literature documents that the most widely used species belong to the families Heterorhabditidae and Steinernematidae, which naturally inhabit the soil and identify their hosts through CO_2_ and other chemical cues emitted by insects [61]. Their pathogenicity toward pest insects is associated with a symbiotic–mutualistic interaction with bacteria of the genus *Xenorhabdus* spp. (specific to *Steinernema* spp.) and *Photorhabdus* spp. (exclusive to *Heterorhabditis* spp.), which are primarily responsible for killing the host insect [62]. In large-scale agricultural programs, EPNs can be mass-produced and applied by spraying using conventional equipment with relative ease [63].

The infective juveniles (IJs) of EPNs penetrate the host hemocoel, where they release a symbiotic bacterium harbored in the nematode’s intestine. Once inside the insect, they induce septicemia, which causes the death of the host within approximately 24–48 h [64]. IJs feed on bacteria that rapidly proliferate and degrade host tissues. The symbiotic relationship between EPNs and bacteria enhances nematode reproduction [45,65]. In this regard, a field study conducted in Oregon, USA, evaluated the efficacy of the EPNs *Steinernema carpocapsae* (Weiser, 1955) and *Heterorhabditis bacteriophora* Poinar, 1976 for the management of the strawberry crown moth, *Synanthedon bibionipennis* (Boisduval, 1869) (Lepidoptera: Sesiidae). Applications performed in late autumn resulted in mortality rates of 51 and 33% for *S. carpocapsae* and *H. bacteriophora*, respectively. The authors further noted that control efficacy could be improved when EPNs are applied in late summer (October), coinciding with the presence of larval stages in the soil, which exhibit increased susceptibility to nematode infection [65]. This highlights a knowledge gap and underscores the need for new EPN-based IPM studies in berries, particularly in developing countries where excessive and irrational amounts of synthetic pesticides are still widely applied [43,66]. EPNs have the advantage of being mass-produced either through in vitro procedures [67] or in vivo. For the latter, the greater wax moth *Galleria mellonella* (Linnaeus, 1758) (Lepidoptera: Pyralidae) is of considerable utility [68]. Nevertheless, although EPNs are highly effective for the natural regulation of insect pests and exhibit great potential in crop protection programs, studies under laboratory and field conditions in berry crops remain scarce (Table 1).

However, to avoid potential failures in the application of EPNs under different conditions (field or greenhouse), it is essential to select the ideal nematode species or strain (preferably native isolates adapted to the environmental conditions of the target areas) [71]. In addition, it is necessary to analyze the optimal characteristics for their use (e.g., desiccation, temperature, UV radiation tolerance, and virulence), which may lead to more effective management of a particular pest species [72,73]. It is also critical to consider that their application should preferably be carried out before sunrise or at sunset, due to their high susceptibility to environmental conditions [74]. Furthermore, it is vital to assess the optimal temperature range for the selected EPN isolates, which is usually between 20 and 30 °C [73]. Taking these factors into account, the main application strategies of EPNs are as follows:

Foliar application. This method consists of inundative application of these agents, emulating chemical control. It has been reported that this approach may be compatible with the simultaneous use of chemical pesticides [75]. Some studies indicate synergistic effects when combined with acetamiprid, spinetoram, malathion, abamectin, azadirachtin, deltamethrin, lambda cyhalothrin, and phosmet, respectively [76]. These pesticides have been applied to berries worldwide, although certain authors report negative effects on beneficial entomofauna [77,78,79]. Other authors combined the application of *H. beicherriana* Li, Liu, Nermuť, Půža & Mráček, 2012 (at a dose of 1 × 10^3^ IJs/plant) with *Bacillus thuringiensis* Berliner, 1915 at a concentration of 1.14 × 10^10^ CFU/plant, achieving mortality rates of 83.9 ± 0.82% in larvae of the white grub *Holotrichia parallela* (Motschulsky, 1854) (Coleoptera: Scarabaeidae) in peanut crops [80]. Such results could also potentially be applied to berries crops in the future.

Drip irrigation. This strategy has been employed in some studies; however, it has been documented as one of the least efficient due to poor distribution of EPNs and uneven application, with nematode sedimentation rates exceeding 34% in irrigation systems of this type [81,82]. This system has been used for irrigating strawberry [83], raspberry [84], and blackberry [85], respectively, although without the addition of EPNs through irrigation.

Aerial application. EPNs can be applied using aerial equipment, including small pressurized sprayers [86]. It has been reported that EPNs can withstand application pressures of up to 300 psi through nozzles with openings of 50 μm in diameter. However, some types of aerial spraying equipment generate high levels of heat, and if the temperature within the sprayer pipes exceeds 32 °C, EPN viability may be negatively affected [87]. Although helicopters are primarily employed in this strategy, in strawberry crops the use of robots or drones could be implemented, adopting a precision agriculture approach with the aim of optimizing EPN application, as has been demonstrated in other studies [88,89].

**Figure 2:**
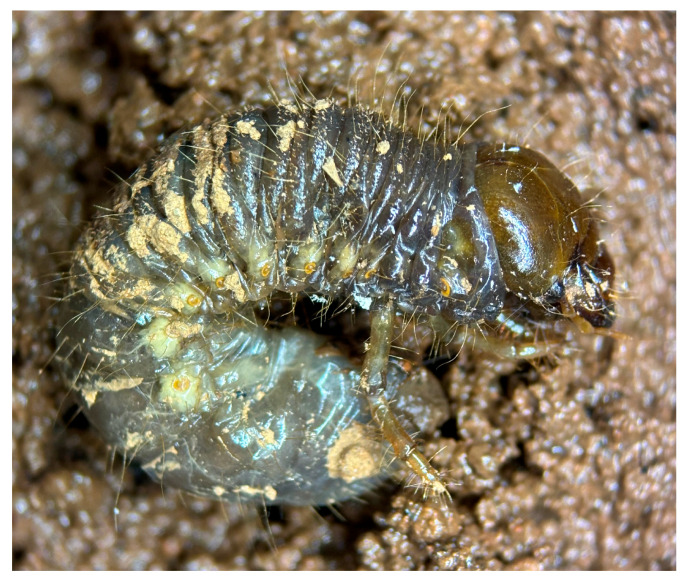
*Phyllophaga* spp. larva infected by the entomopathogenic nematode *Heterorhabditis* spp. Image courtesy of author G.A.-R.

Inoculation through infected cadavers. This may represent the most suitable EPN application approach in berries, particularly in developing countries, since the inoculative scheme has demonstrated that IJs emerging from infected cadavers disperse more efficiently and exhibit higher virulence. Consequently, this application technique may even surpass the inundative spraying strategy (previously described under foliar and aerial application methods) [90,91]. This method has been employed in other crops under controlled (greenhouse) conditions. For example, in cucumber, the potential of *Heterorhabditis* sp. was tested for the biological control of the banded cucumber beetle *Diabrotica balteata* LeConte, 1865 (Coleoptera: Chrysomelidae) and the root weevil *Diaprepes abbreviatus* Linnaeus, 1758 (Coleoptera: Curculionidae). Results indicated that infected cadaver applications achieved efficacy rates exceeding 95% [92]. Despite these successes, infected cadavers have not been widely utilized, leaving a research field that still holds great potential to contribute to scientific knowledge in strawberry production. Figure 2 shows a *Phyllophaga* spp. larva with mortality associated with *Heterorhabditis* spp., which demonstrates the potential of this technique.

All these entomopathogens together can be applied in agroecosystems through various techniques such as flood spraying [93,94,95] and soil application [82,96,97] and to contribute to CBC through the strategy of self-dissemination (which consists of manipulating the behavior of pest insects to favor the dissemination of entomopathogens to their susceptible conspecifics) [98], representing the basis for their permanence in agroecosystems. Based on the above, recent studies have examined the use of NEs (predators, parasitoids, and pollinators) as dispersers of entomopathogens under laboratory and field conditions, thus combining micro- and macrobiological control strategies [17,99,100,101,102].

Currently the worldwide use of pesticides and entomopathogens is based on their application by the spraying technique, and the disadvantage is that it is one of the least efficient [103]. It has been documented that approximately 1% of the active ingredient from chemical pesticides actually reaches the target insect [104], so the remaining 99% ends up in the environment or non-target organisms, such as predators, parasitoids, and pollinators, which could lead to up to 40% extinction in the next few years [105,106]. So it is necessary to consider that the entomopathogens to be used present a high virulence [107] although, in the CBC paradigm, the objective is to cause epizootics in the pest insect populations. To achieve this, rigorous studies must be conducted that consider ecological and epidemiological aspects and analyze the biotic and abiotic factors that allow the development of new formulations of biological insecticides using these agents as active ingredients. Failure to take these variables into account has led to a lack of confidence in the use of these agents for extensive application in agroecosystems worldwide, as reported in interesting review articles [87,108,109].

## 4. Physiological and Ecological Determinants of BVs, EPFs, and EPNs in Berry Pest Management

It is necessary to consider that, for BVs, EPFs, and EPNs to thrive, environmental factors that are highly crucial in the physiology of these agents must be taken into account; mainly temperature, humidity, and UV radiation [35,36,110]. In this sense, the inactivation of *Baculovirus heliothis* by artificial UV irradiation applied by spraying at a concentration of 1 × 10^8^ polyhedral inclusion bodies (PIBs) on cotton (*Gossypium hirsutum* L., 1763) and soybean (*Glycine max* (L.) Merr., 1917) plants [111] showed that inactivation was directly related to the period of exposure. Thus, the losses of activity for virus exposed between 0 and 12, 12 and 24, and 24 and 48 h were 78.9, 86.9, and 84.1%, respectively. Viruses not exposed to UV radiation did not show statistically significant losses (*p* = 0.0001) of activity, while in some studies the exposure of *B. bassiana* and *M. anisopliae* conidia after receiving UV radiation at 5–30 min intervals has been evaluated, finding that radiation had a deleterious effect after the first 5 min of exposure, where germination dropped from 94 to 52% for *B. bassiana* and from 96 to 64% for *M. anisopliae*, respectively [112]. In the case of EPNs, a study by Gulzar et al. [94] compared the tolerance to UV radiation (254 nm) for 10–20 min of exposure and virulence on *G. mellonella* larvae of nine different species of EPNs: *H. bacteriophora*, *H. floridensis* Nguyen, Gozel, Köppenhöfer & Adams, 2006, *H. georgiana* Nguyen, Shapiro-Ilan & Mbata, 2008, *H. indica* Poinar, Karunakar & David, 1992, *H. megidis* Poinar, Jackson & Klein, 1987, *S. carpocapsae*, *S. feltiae* (Filipjev, 1934), *S. rarum* (Doucet, 1986), and *S. riobrave* Cabanillas, Poinar & Raulston, 1994. The results obtained revealed that *Steinernema* spp. showed superior tolerance to UV compared to *Heterorhabditis* spp.; furthermore, all Heterorhabditidae showed reduced fitness after 20 min of exposure, although Steinernematidae did not. In terms of virulence, 48 h after infection, all EPN species showed a significant reduction (60% for *Steinernema* spp. and 10% for *Heterorhabditis* spp.).

In general, and for the correct functioning of these entomopathogens, they must be applied when there are large populations of insects so that the spraying has the desired effects, since the delayed mortality rate is one of the reasons why producers do not consider their use [113]. In addition, it is necessary to take into account the functioning of the BV occlusion bodies (OBs), which orally enter the target insect and dissolve in its alkaline gut (which has a pH varying between 10 and 12) [114]; while, in the case of EPFs, it is necessary to take into account the speed at which conidia germinate [115] and, finally, in the case of EPNs, proper storage, handling, formulation, and application are fundamental for their successful use in biological control [116,117]. Consideration of these issues could greatly enhance the results during the application of these biocontrol agents.

## 5. Predatory Arthropods in Berry Production: New Strategies and Current Challenges

Predatory mites, mostly Phytoseiid mites (Acari: Phytoseiidae), are widely used in agriculture due to their diverse feeding habits, ability to establish in a wide range of crops, and pest suppression of several phytophagous arthropods. According to their feeding habits they are classified in four different types (Table 2).

**Table 2:** Classification of Phytoseiid mites based on their feeding habits [118].

Type	Feeding Habits	Species Example	Prey
I	Highly specific predators	*Phytoseiulus persimilis*	*Tetranychus* spider mites
II	Selective predators	*Neoseiulus californicus*	Phytophagous mites
III	Generalist predators	*Amblyseius swirskii*	Primarily predators of a wide range of arthropods (mites, thrips, whiteflies, psyllids, etc.). Occasional pollen feeders (Figure 3)
IV	Pollen feeders	*Euseius* sp.	Primarily pollen feeders, occasional generalist predators

**Figure 3:**
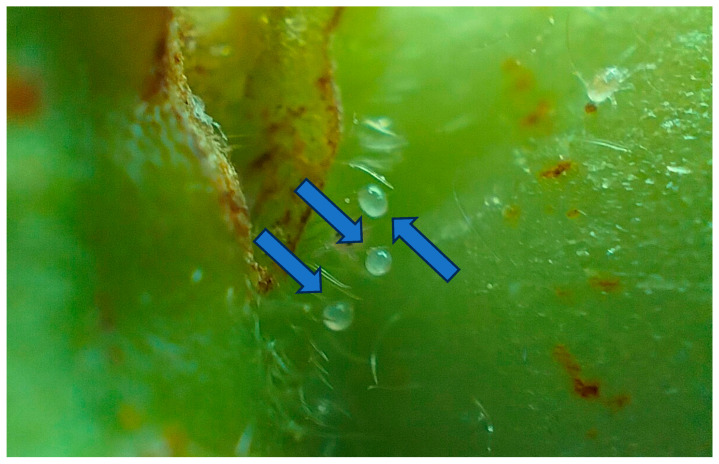
*Amblyseius swirskii* eggs on a strawberry leaf. Image courtesy of author G.A.-R.

A recent classification proposes dividing phytoseiids into type I and type III subgroups, based on their preferred prey type and microhabitat, respectively [118]. These features highlight the complexity and diverse behavior exhibited by phytoseiids which have allowed their incorporation into different strategies of IPM across multiple countries, crops, and agricultural systems. Furthermore, these arthropods are commercialized in a wide range of countries by several companies like Koppert^®^, Biobest^®^, Bioline^®^, and BioBee^®^, just to mention a few.

**Figure 4:**
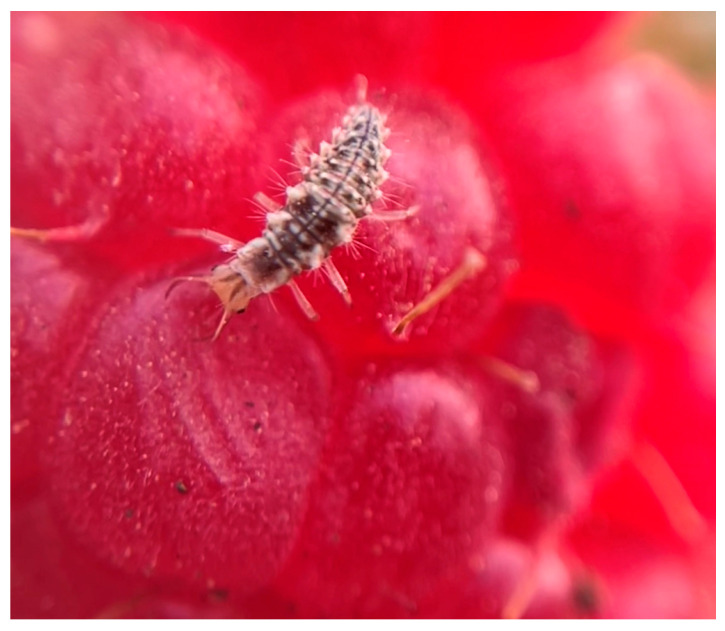
*Chrysoperla carnea* L_3_ larva (<24 h after molting) in blackberry crops. Image courtesy of author G.A.-R.

**Figure 5:**
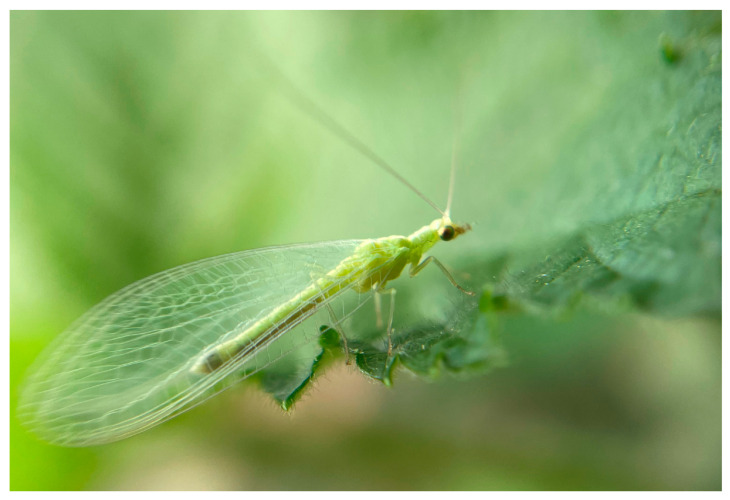
Adult male of *C. carnea* positioned on a blackberry leaf. Image courtesy of author G.A.-R.

Chemical pest control can become progressively less effective over time due to the development of resistance resulting from the repeated and intensive use of specific active ingredients. For example, *T. urticae* is one of the most economically significant pests in strawberry production, causing yield losses of up to 25% of marketable fruit [119]. As management of this species has relied predominantly on abamectin-based acaricides, multiple cases of resistance have already been documented [120,121,122]. This scenario has driven the adoption of alternative control strategies, including the use of natural enemies such as predatory mites and insects. Among these, *Chrysoperla carnea* (Stephens, 1836) (Neuroptera: Chrysopidae) has received considerable attention, as its larval stage functions as a generalist predator of insect pests (Figure 4), whereas adults primarily feed on pollen, nectar, and honeydew (Figure 5).

In the specific case of *T. urticae* in strawberry, releases of *N. californicus* and *P. persimilis* (type II and I phytoseiids, respectively) can offer long-term control of this pest [123]. Moreover, while the initial cost of releasing predatory mites might seem higher than chemical sprays, the long-term economic benefits are superior. A study comparing *N. californicus* and a chemical acaricide on strawberry found that the predatory mite release was the more competent and effective tactic in long-term suppression of *T. urticae* [123]. Similarly, different studies have shown that the release of *N. californicus* and also the type III phytoseiid *Neoseiulus cucumeris* (Oudemans, 1930) (Mesostigmata: Phytoseiidae) can effectively suppress the cyclamen mite *Phytonemus pallidus* (Banks, 1899) (Trombidiformes: Tarsonemidae) and increase the marketable fruit-class yield by 86% [40,124].

Recent research has shown that the establishment of phytoseiid mites could also be supported by incorporating “banker plants” that will provide a self-sustaining food source and habitat for predatory mites and other beneficials. For example, while it is known that tomato provides a harsh environment for phytoseiid mite establishment [125], a recent study has shown that the incorporation of *Mentha suaveolens* Ehrh., 1792 in tomato production enhances the establishment of *Typhlodromus recki* Wainstein, 1958 (Mesostigmata: Phytoseiidae) and facilitates the pest suppression of the russet mite *Aculops lycopersici* (Tryon, 1917) (Trombidiformes: Eriophyidae) [126]. In strawberries, *Lobularia maritima* Desv., 1815 (sweet alyssum) and *Capsicum annuum* L., 1753 (ornamental pepper) are great options to increase and support the establishment of beneficial arthropods, resulting in the effective control of *Scirtothrips dorsalis* Hood, 1919 (Thysanoptera: Thripidae) [127]. Although this strategy seems a promising tool within IPM strategies, further research is required in field evaluations of blueberry, raspberry, and blackberry crops.

While there are several reports of compatibility of EPFs with predatory mites, selecting the right features of an EPF, such as strain specificity, is critical to ensure the synergy of both organisms within the IPM program. Some of them have shown high susceptibility of the mite *Amblyseius swirskii* Athias-Henriot, 1962 (Mesostigmata: Phytoseiidae) to the EPF *B. bassiana* [128]; while others, in contrast, showed that some strains might be harmless to several species of phytoseiid mites [129]. In some cases, selecting the right EPF and predatory mite species might result in greater pest suppression which might be greater when combining them compared to stand-alone treatments [130]. In blueberry production the chilli thrips *S. dorsalis* remains as one of the main challenges in the crop. So far, there are no reports of successful control of this pest in the mentioned crop using predatory mites. This may be attributed to establishment difficulties that commercially available species may exhibit. In fact, a recent study showed that, among raspberry, blackberry, and blueberry, the latter presented the lowest phytoseiid diversity [131]. Although this challenging scenario might seem discouraging, given the global importance of blueberry production, it also endorses research and industry to develop and investigate further options for the biological control of chilli thrips in this crop.

Another major challenge of incorporating predatory mites into IPM systems is the incompatibility with certain insecticide/acaricide molecules. For instance, a study showed that malathion and bifenthrin are extremely harmful for *N. cucumeris*, even at low doses [132]. Moreover, some insecticides such as neonicotinoids are known to be very persistent, remaining more than 30 days in the crop [133]. Thus, releasing predatory mites after spraying the mentioned molecules (or vice versa) will result in the failure of mite establishment, higher costs, and poor pest suppression (if resistance is involved). Thus, selecting compatible molecules is also critical for the success of biological control programs. Other authors suggest that bifenazate may be compatible with predatory mite releases if it is applied localized in *T. urticae* hotspots [134]. Similarly, azadirachtin may also be considered as a complementary and compatible tool with predatory mites for the IPM of *T. urticae* [135]. The use of predatory mites in conventional agricultural systems is constantly improving as new techniques and methods for the assessment of compatibility also improve [136]. This highlights the importance of constant extension and education on this matter to ensure the proper use of predatory mites within IPM. Moreover, extending these practices to growers will keep contributing to the decrease in highly residual insecticides/acaricides.

Other predatory insects have also shown promising results for pest suppression in berry production. For instance, *Orius* spp. have demonstrated promising results for the suppression of *Scirtothrips* sp. in strawberry [137]. Similarly, green lacewings were also able to suppress aphid infestation in strawberry [138]. A recent study also showed the great potential of brown lacewings *Micromus angulatus* (Stephens, 1836) (Neuroptera: Hemerobiidae) as an agent for aphid control [139]. Nevertheless, most of the mentioned research has been performed in strawberries, and thus the behavior and potential of these biological control agents in other crops like blueberries, blackberries, and raspberries remain relatively unexplored. Moreover, the impact of native and naturally occurring biocontrol agents on these crops remains poorly studied.

## 6. Wasps as an Essential Part of IPM: The Case of Spotted Wing Drosophila (SWD)

The spotted wing drosophila, *D. suzukii* (SWD), is among the most economically important pests affecting berry production worldwide [140]. Management of this species has traditionally relied on intensive chemical applications [141]; however, the sustained use of insecticides in the absence of complementary control strategies within an IPM framework poses substantial long-term risks. Notably, resistance to spinosad in SWD populations has already been documented in California [142], underscoring the urgent need for alternative and sustainable control approaches. In response, the incorporation of parasitoid wasps has emerged over the past decade as a promising biological control strategy for SWD.

Candidate parasitoid A. Recent studies have identified the larval parasitoid *Ganaspis kimorum* Buffington, 2024 (Hymenoptera: Figitidae) as a potential biological control agent of SWD, largely due to its initially reported narrow host specificity [143]. This species was originally considered part of *Ganaspis brasiliensis* (Ihering, 1905) (Hymenoptera: Figitidae) until molecular and taxonomic analyses revealed that *G. brasiliensis* comprised multiple cryptic genetic lineages [144]. Subsequent research demonstrated that these lineages differed in behavioral traits and host range [145], and more recent evidence confirmed that several of these lineages represented distinct species [146]. Consequently, *G. kimorum* (G1) and *Ganaspis lupini* (Ihering, 1905) (Hymenoptera: Figitidae) (G3) were formally recognized as separate species within the *G. brasiliensis* species complex.

Despite its promise, *G. kimorum* exhibits a broader host range than initially assumed, which may reduce its suitability for classical biological control programs targeting SWD exclusively. This case highlights the critical importance of accurate taxonomic identification and ecological characterization of parasitoids to ensure effective and targeted pest suppression within IPM programs. Additionally, a major limitation of *G. kimorum* is its poor performance on artificial diets, which complicates mass-rearing protocols and restricts its application in augmentative biological control. Further research is required to overcome these rearing constraints and to determine optimal release strategies, including dosage, frequency, and cost-effectiveness, under field conditions. Nevertheless, *G. kimorum* remains one of the most promising parasitoids for SWD management within IPM frameworks.

Candidate parasitoid B. The pupal parasitoid *Trichopria drosophilae* (Perkins, 1910) (Hymenoptera: Diapriidae) has been extensively studied in recent years and consistently demonstrates high potential as a key component of SWD IPM programs [147,148]. Although *T. drosophilae* has a broader host range compared with *G. kimorum*, it offers significant practical advantages, particularly its relatively simple and efficient mass-rearing under laboratory conditions. This characteristic facilitates large-scale production and supports its use in augmentative release programs in commercial berry systems.

However, a major limitation associated with *T. drosophilae* is its limited commercial availability, as only a small number of biological control companies currently offer this parasitoid. To address this constraint, some berry-producing enterprises have established in-house rearing facilities to ensure a consistent supply of biological control agents. For example, reiter affiliated companies developed their own beneficial insect production unit, Biological Farming Solutions (BFS), which currently mass-produces and releases more than six million parasitoid wasps annually across strawberry, raspberry, blackberry, and blueberry crops in multiple regions of Mexico and the United States (personal experience/communication). As a result, augmentative releases of *T. drosophilae* have become a standard and, in some cases, mandatory component of SWD IPM programs for a substantial number of commercial growers.

## 7. Perspectives

Yet they remain particularly vulnerable to diverse arthropod pests due to their low genetic variability and intensive monocultural systems. Conventional pest control strategies based on synthetic pesticides, although effective in the short term, have generated resistance, environmental damage, and regulatory restrictions for export markets. In this context, the integration of microbiological agents such as BVs, EPFs, and EPNs together with macrobiological control agents, including predators and parasitoids, emerges as a sustainable alternative to mitigate pest pressure and promote food security [149,150,151,152,153,154]. Advances in genetic engineering of entomopathogens have further expanded their efficacy, specificity, and potential for mass production, offering novel tools for biological control under both field and greenhouse conditions. In recent years, the use of EPFs has resurged in developed countries. However, in developing nations such as those in Latin America, their large-scale field application remains limited [23]. One of the main challenges is the correct formulation, handling, and application of these fungi. Another important consideration is the cost of production and their methodology limitations, techniques, and specialized human resources for production and application directly by farmers, for instance, in the case of *M. anisopliae* and *M. acridum* massive production was supported by government programs in Brazil, Perú, Colombia, Mexico, and Argentina [155,156]. But is necessary to reduce limitations and gaps in public policies that enable the proper implementation, regulation, and management of the IPM [157,158].

Moreover, some EPF strains display only partial efficacy or lack broad-spectrum activity. Although EPFs produce a wide variety of metabolites—including non-ribosomal peptides, polyketides, beauvericin, bassianolide, beauverolides, oosporein, bassiatin, and tenellin (2-pyridone)—not all are synthesized at effective concentrations [21,159,160,161], which would represent a highly promising approach for application in berry cultivation, where international export regulations require that these products be free of synthetic pesticide residues in order to ensure their safe consumption [162].

Recent advances in genetic engineering have facilitated the development of EPFs with enhanced virulence and efficacy against pests [55,163]. For instance, the CRISPR-Cas9 system was employed to edit the *Bbsmr1* gene, which regulates oosporein biosynthesis, resulting in overproduction levels reaching up to 118 ppm after four days of fermentation [164]. In *M. brunneum*, genetic modification of the *dmaW*, *easF, easC*, *easE*, and *easD* genes—key elements in lysergic acid (LA) and dihydrolysergic acid (DHLA) biosynthetic pathways—has been pursued. These compounds are valuable precursors for pharmaceutical semi-synthesis, although they frequently occur as transient intermediates in ergot alkaloid pathways [165]. Similarly, some authors have reported the successful transformation of *M. anisopliae* with the *LqqIT1a* gene, significantly increasing virulence against *Spodoptera litura* (Fabricius, 1775) (Lepidoptera: Noctuidae) and *Aphis craccivora* Koch, 1854 (Hemiptera: Aphididae) [21], both insects being important pests of strawberry crops [166,167]. Transformed strains killed their hosts in 1.12 days, compared with 4.37 days required by unmodified strains [21,109]. Likewise, *Aspergillus nomiae* B.W. Horn, I. Carbone & G.G. Moore, 1987 strains modified as AnS1Gz1-1 expanded their infection spectrum to Lepidoptera and Hemiptera.

Beyond insect control, genetic engineering has also been used to enhance EPF–plant interactions by activating key defense pathways, including salicylic acid (SA) and jasmonic acid (JA) signaling [38,168], which demonstrated that genetically modified EPF strains can induce plant defense activation [62,73,168]. The main modifications involve genes encoding enzymes such as chitinases, lipases, and proteases, as well as secondary metabolites including beauvericin, isoleucyl anhydride, cyclo-(L-isoleucyl-L-valine), cyclo-(L-alanyl-L-proline), bassianin, oosporein, bassiacridin, bassianolide, beauveriolides I and III, 5-hydroxypiperlongumine, chrysazine, globosuxanthon A, pyridovericin, beauvetetraones A–C, and dipicolinic acid. These metabolites hold relevance across agriculture, nutritional biotechnology, industrial biotechnology, and environmental protection [26,169]. Some limitations exist in the massive production and application of EPFs in open fields, for example, climatic and ambient adaptations in some isolated fungal strains highlight the need to isolate and characterize local EPFs that already have adaptive characteristics and also use them as a reference to identify genes that confer such adaptation [170,171].

Looking forward, the outlook for EPFs points to the continuous improvement of strains for enhanced secondary metabolite production through the combined use of CRISPR-Cas9 technologies and blastospore-based mass production, enabling concentrations suitable for agricultural and pharmaceutical applications [172,173]. The integration of genetic engineering strategies is thus expected to yield new EPF strains capable of meeting industrial demands in a rapid and cost-effective manner [174,175]. Even with everything described above, their successful application requires careful consideration of ecological, physiological, political, social, and environmental factors, particularly temperature, humidity, and UV radiation, which directly influence their persistence and virulence. It is also important to consider biological control focused on berries as an effective IPM tool. Furthermore, it should be noted that it is a modification of existing practices (the current paradigm of agricultural production based on chemical inputs) aimed at protecting NEs, beneficial entomofauna species, and specifically other organisms to reduce the effect of pests [17]. In short, biofactories need to incorporate local genetically modified EPF strains to improve their virulence and increase their efficacy [164].

In addition, the importance of pest control management in strawberries lies not only in the conservation of entomofauna but also in reducing post-harvest residues on fruits. Berries have recently received attention regarding their nutritional value; however, high concentrations due to resistance and poor agricultural practices are an important consideration when it comes to consumption [176]. Therefore, this section invites us to question alternatives to biological control or good agricultural practices, but also suggests a change in the entire paradigm of food production, a paradigm that faces challenges in scientific research.

## 8. Conclusions

Moreover, strategies such as conservation biological control and the optimization of application methods have the potential to strengthen the long-term impact of natural enemies within agroecosystems, for instance, genetic engineering approaches. While encouraging progress has been documented in developed countries, widespread implementation in developing regions remains limited, highlighting the urgent need for applied research, effective technology transfer, and social and political programs tailored to each country’s specific context. Together, the integration of engineered macro- and microbiological control agents represents a promising and sustainable alternative to synthetic pesticides, promoting safer fruit production systems and contributing to broader goals of environmental sustainability and global food security.

## Figures and Tables

**Table 1:** Entomopathogenic Nematode Species and Their Role in the Regulation of Berry Pest Insects Worldwide.

EPN Species	Crop	Target Pest	Reference ^1^
*H. marelatus* Liu & Berry, 1996	Strawberry	*Otiorhynchus sulcatus* (Fabricius, 1775) (Coleoptera: Curculionidae)	[11]
*S. carpocapsae* and *S. glaseri* (Steiner, 1929)	Blueberries, strawberries, and raspberries	*O. sulcatus*	[69]
*S. carpocapsae* and *H. bacteriophora*	Strawberry	*Synanthedon bibionipennis* (Boisduval, 1869) (Lepidoptera: Sesiidae)	[65]
*H. bacteriophora*	Strawberry	*Lobiopa insularis* (Laporte de Castelnau, 1840) (Coleoptera: Nitidulidae)	[70]

^1^ It is written in chronological order.

## Data Availability

No new data were created or analyzed in this study. Data sharing is not applicable to this article.
